# Telling lives in medicine: the impact of biography collections in medical education

**DOI:** 10.5195/jmla.2021.1312

**Published:** 2021-07-01

**Authors:** Sofia Fagiolo

**Affiliations:** 1 s.fagiolo@unicampus.it, Cataloging Librarian, Library, Campus Bio-Medico University of Rome, Italy

**Keywords:** medical biographies, biographies collections, medical education, medical humanities, academic medical libraries

## Abstract

This article briefly discusses the value and impact of biography collections in medical education by illustrating the case of the Campus Bio-Medico University of Rome (UCBM) Library. The UCBM Library collects, curates, and provides access to a special biography collection with the purpose of documenting the history of men and women who contributed in the field of medicine and related sciences. This article highlights the importance of academic medical libraries collecting biographical works in order to transmit knowledge and values in medical school curriculum.

## INTRODUCTION

In recent years, medical humanities has increasingly attracted attention, and many universities have added medical humanities programs to their medical schools with the purpose of training more insightful and empathetic doctors [1, 2]. In this regard, medical biographies can play a captivating role, enriching the experiences of students. Several studies have stressed the didactic role of biographies in the effort of humanizing medical practice [3].

Over time, the Campus Bio-Medico University of Rome (UCBM) Library has built up a sizeable collection of medical and scientific biographies for educational purposes. This collection enhances the educational experience on one hand and promotes the institutional mission on the other. The following is an overview of the history of the university, a description of the biographies collection, and its educational role among medical students.

## HISTORY

UCBM established itself in 1993 as a medical university inspired by the Christian value of service in alleviating human suffering and advancing knowledge and understanding in health care. UCBM is a small (approximately 1,800 students) private institution with undergraduate and postgraduate courses in medicine and surgery, engineering, and food science. Since its beginning, the university has worked to achieve excellence in medical and health education, research, and patient care, adhering to the highest ethical values. Humanizing medicine and care is the mission of most programs at UCBM. Courses and seminars focus on the importance of doctor-patient relations, medical ethics, and cross-cultural understanding [4].

**Figure 1:**
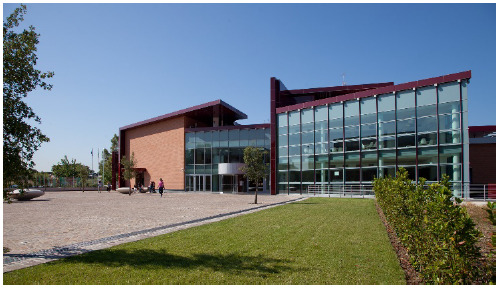
The “Trapezio” building of the university with an external view of the library, which is housed in the glass building on the right side. Photo credit: Campus Bio-Medico University of Rome

The UCBM Library (Figure 1) is committed to support and enhance the teaching, education, and research activities of the university by providing materials and services to students, researchers, and faculty. In particular, it provides valuable sources for the study of medical history by collecting, preserving, and providing access to special book collections related to the history of medicine. By doing so, the library contributes to a better understanding of the historical and cultural context of medicine, matching the educational objectives of the institution.

Among its special collections, the library holds an extensive collection of biographical and autobiographical accounts of people who advanced medicine and related fields. Inspired by the teaching of William Osler (1849–1919), in 2009 the library started to collect biographical writings of medical and scientific figures of the past, with the hope of designing a collection that would be a source of inspiration and motivation for students. Osler's appreciation for medical biographies is well known; he believed that “medical biography could inspire young members of the profession to envision bright possibilities for their lives” [5]. Medical biography is a very old genre; however, until the early twentieth century, it did not have as strong a presence as its sister genre, the scientific biography [6]. Osler is indeed recognized for giving a great impetus to the study of medical history through a “biographical” perspective [7].

The goal of the biography collection at the UCBM Library is to highlight the human aspect of medicine through the real experience of individuals. For this reason, this collection is an integral part of the teaching of the university's programs and seminars with a strong humanistic orientation.

## THE COLLECTION

The biography collection at UCBM Library contains more than 1,200 titles covering a wide range of sources, including biographies, autobiographies, memoirs, diaries, speeches, epistolary books, obituaries, and biographical dictionaries and encyclopedias. Rare and old books are also present in this collection, some of which are very valuable. The main languages covered are Italian and English, but there are also holdings in other languages including French, Spanish, German, and Portuguese.

**Figure 2:**
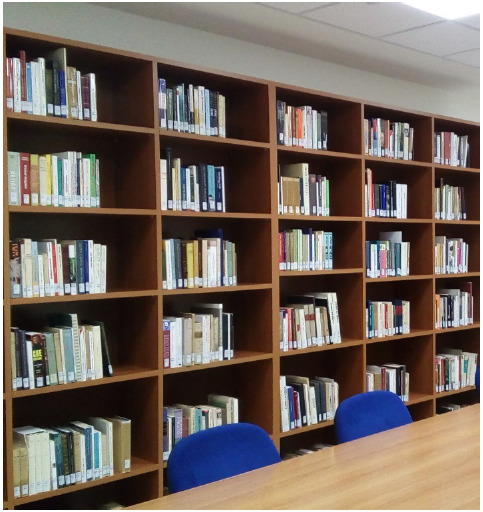
Part of the Biographies Room. Photo credit: Campus Bio-Medico University of Rome

All these books are shelved in a separate room designated as the Biographies Room (Figure 2). Individual biographies are classified in the Dewey call number 920 (“Biography, genealogy, insignia”), followed by the last name of the person written about: for example, “920 Laennec” is a biography about René Laennec. This process enables titles to be arranged alphabetically according to the biographee's last name. Collective biographies and biographical dictionaries and encyclopedias are shelved after the individual biographies and are classified under the call number 920.003 (“Dictionaries, encyclopedias, concordances”). Collective biographies with a group of people associated with a specific field (e.g., biography of surgeons) are arranged according to the attribute of these people (e.g., “surgeons”). Pamphlets and brochures are stored separately in folders.

Like the other collections present in the library, the biography collection is divided into circulating and noncirculating materials on the basis of the publication date. Books published before 1945 are not loaned. Access to the Biographies Room is currently for internal library use only due to COVID-19 restrictions, but in normal times students usually enter the room to consult the books. To help students find biographical resources available to them, a special section in the library website was created. In this section, each biographical work is provided with brief information on the biographee, with a link to a Wikipedia page.

The collection covers not only notable people and Nobel Prize winners but also lesser well-known people whose lives and works students know little. Furthermore, it preserves the memory of unknown medical scientists and researchers thanks to the presence of a modest collection of academic obituaries and memorial articles extracted from scientific journals. The collection does not limit itself to any chronological periods, although the majority of the titles focus on the period from the eighteenth to twentieth century.

The biography collection covers a wide array of disciplines; students can find in this collection not only the biographies of health care providers but also the lives and stories of biologists, explorers, naturalists, biochemists, physicists, mathematicians, philosophers, psychologists, and religious leaders who made a mark in health care and assistance services. Since UCBM has a Faculty of Engineering, the collection also holds several biographical works devoted to men and women who contributed to the advancement of engineering and, of course, frontier lives between medicine and engineering. In particular, it contains the stories of protagonists from the fields of surgery and engineering who pioneered the technological advancement of cardiac surgery [8].

## MEDICAL BIOGRAPHIES AS EDUCATIONAL SOURCES

The medical-biographical literature constitutes an excellent and fascinating way to engage students' interest in the cultural and historical basics of their new profession. Moreover, biographies can give medical students a new understanding and sense of encouragement for their studies.

Holding a biography collection is not enough, however; it should be linked to the university's life. A meaningful way to reach this goal is to incorporate biographies in academic programs, putting them to work for the medical curriculum. At UCBM, biographies are routinely used for the History of Medicine program. To pass their exam in this course, students are asked to read and discuss the biography of a particular medical/health care figure of their choice. Titles are selected from the biography collection with the assistance and guidance of librarians.

The main goal of this biographical approach is to encourage students to learn more about their future profession through a humanizing dimension. By reading the personal drama, success, and failure of medical men and women, students acknowledge the many facets of being a physician. They discover the trials and challenges these men and women experienced through their lives and professional careers and learn that perseverance and resilience are keys to success. Furthermore, the experience of these medical figures can help students to reflect on deeper questions regarding their professional identity and role in society. Biographies provide insights into the lives of these personalities that can be very inspiring, so students can find in these stories the moral foundations necessary to guide them in their future profession [9].

Another aim of this educational approach is to encourage students to develop an interest in the humanities. Since biographies reveal the human side of medicine, with its social, cultural, and historical implications, they are inextricably linked to the intersection of medicine and humanities, supporting the exploration of disciplines such as history, philosophy, sociology, politics, religion, literature, and art. The History of Medicine program puts special emphasis on this aspect, which is why in the biography collection there are many works about physician writers, musicians, and artists.

## CONCLUSION

Reading about the medical lives and careers of men and women of the past is beneficial for the professional formation of medical students. Biographies, especially when integrated into academic programs, can foster conversation around issues such as the social responsibility of medical providers, the importance of a moral and scientific integrity, the ethical implications of the professional practice of medicine, as well as interest in the humanities.

By preserving and providing access to a biography collection, the UCBM Library fulfills the educational goals of the institution in the promotion of the social, cultural, and ethical aspects of medicine.
